# Expression Profiling of Reelin (RELN) Gene in Patients With Schizophrenia from North Karnataka

**DOI:** 10.7759/cureus.76850

**Published:** 2025-01-03

**Authors:** Nilima Dongre, Chetan Shattar, Santosh Ramdurg, Gurushantappa S Kadakol

**Affiliations:** 1 Biochemistry, BLDE (Deemed to be University) Shri B M Patil Medical College, Hospital and Research Centre, Vijayapura, IND; 2 Psychiatry, BLDE (Deemed to be University) Shri B M Patil Medical College, Hospital and Research Centre, Vijayapura, IND; 3 Anatomy, Human Genetics Laboratory, BLDE (Deemed to be University) Shri B M Patil Medical College, Hospital and Research Centre, Vijayapura, IND

**Keywords:** expression analysis, glucose level, lipid profile, reln gene, rt-pcr, schizophrenia

## Abstract

Background

Schizophrenia is a devastating neuropsychiatric condition of uncertain etiology with significant adverse effects on the affected people, their families, and society. India has a heterogeneous population with a high degree of inbreeding. Hence, it is necessary to screen Indian patients with psychotic disorders to get a true picture of the contribution of Reelin (RELN) mRNA expression in schizophrenia. Mental illness is a leading cause of several metabolic changes and other related complications. It is not clear whether these metabolic changes are due to alterations in the RELN gene expression or because of antipsychotic drug use. Therefore, it is necessary to study the link between RELN gene expression and the metabolic syndrome.

Aim and objective

The present study aims to study the expression profiling of the RELN gene in patients with schizophrenia and the occurrence of metabolic syndrome in these patients.

Methodology

Patients with clinically diagnosed schizophrenia were studied for RELN gene expression and the RELN protein was quantified using real-time reverse transcriptase-polymerase chain reaction (RT-PCR). Biochemical parameters like serum random blood sugar (RBS) and lipid profile were analyzed by standard biochemical methods on the semi-auto analyzer and the lipid ratios in the study population were calculated and compared with the age and sex-matched controls. The statistical analyses were performed using IBM SPSS Statistics for Windows, Version 20 (Released 2011; IBM Corp., Armonk, New York, United States). A p-value of <0.05 was considered statistically significant. All statistical tests were two-tailed.

Results

The present study involved 108 subjects, out of which 54 were patients with schizophrenia (study group) and the remaining 54 were healthy controls (control group). In the former group, 29 were female (53.7%) and 25 were male (46.3%) patients whereas in the latter group, 27 were female (50%) and 27 were male (50%) individuals. Majority of the participants in the study group showed moderate scores on the Brief Psychiatric Rating (BPRS) scale. The levels of RELN mRNA expression were decreased in schizophrenia. Compared to the controls, the levels of RBS, total cholesterol, triglycerides (TGs), and low-density lipoprotein cholesterol (LDL-C) were significantly increased and levels of high-density lipoprotein cholesterol (HDL-C) were significantly decreased in the patients with schizophrenia. Body mass index (BMI), waist circumference (WC), and lipid ratios were also significantly greater in these patients.

Conclusion

An analysis of the candidate genes in psychotic disorders can be helpful in designing targeted drugs to treat these patients. Early and regular monitoring of patients on antipsychotic drug treatment is needed to find and prevent the risk of developing metabolic syndrome, which is the major cause for atherosclerotic cardiovascular diseases in this group. Appropriate lifestyle modifications and early intervention can help in preventing early deaths in patients with psychotic disorders.

## Introduction

Schizophrenia translates to “divided mind.” It is found that young adults are mainly affected by this neurodevelopmental disorder. The symptoms primarily include cognitive and emotional disturbances, including negative (such as aversion, alogia, apathy, poor or non-existent social functioning) and positive (such as delusions and hallucinations) symptoms. The World Health Organization (WHO) estimated that 24 million people were affected with schizophrenia [1]. According to the WHO, its age-standardized prevalence per 100,000 ranged from 343 in Africa to 544 in Japan and Oceania for men and from 378 in Africa to 527 in Southeastern Europe for women [2]. According to recent publications, the median incidence of schizophrenia fluctuates between 0.15 and 0.20 per 1000 people per year and is greater (7 per 1000 people) in the age group of 15 to 35 years [3]. Globally, it results in about 1% of disability-adjusted life years [4]. There are significant gender differences among those who have schizophrenia. Men typically exhibit more negative symptoms than women, while the latter show more effective signs.

The diagnostic criteria for schizophrenia fall under the Diagnostic and Statistical Manual of Mental Disorders (DSM) chapter for the Schizophrenia Spectrum and Other Psychotic Disorders Class. The three main categories of symptoms described in schizophrenia are psychotic or positive symptoms, negative symptoms, and cognitive dysfunction. An inadequate knowledge of the origins of schizophrenic disorders is one of the factors contributing to the continuous difficulty in categorizing these disorders. These illnesses are believed to be the outcome of genetic, neurological, and environmental factors. According to a leading neurobiological theory, the condition may also be related to high amounts of dopamine, a chemical in the brain that carries messages (neurotransmitter) [5].

Approximately 80% of the patients were related to the genetic component with an inheritance pattern. The Schizophrenia Working Group of Psychiatric Genomics studied copy number variations and single nucleotide polymorphisms (SNPs) as a complement to schizophrenia genetics in genome-wide association studies. According to some findings, schizophrenia may be caused by multiple functional differences in genes in the neurodevelopmental pathways [6].

RELN is an extracellular matrix glycoprotein involved in neuronal cell migration and lamination of the corticolimbic structures during prenatal development. In addition, it promotes protein translation, dendrite outgrowth, and spine development during early postnatal life. RELN regulates neuroplasticity and functioning in adults and contributes to the development of the hippocampal neurons [7]. RELN activates the very low-density lipoprotein receptor (VLDLR) and apolipoprotein E2 (APOE 2), which have physiologic repercussions. The RELN signal also activates Src/Fyn kinases (Dab1) and phosphorylates the adaptor protein Disable-1. RELN may play a role in the emergence of neurodevelopmental diseases such as schizophrenia. Thus, potential genes for schizophrenia include genes encoding RELN and proteins implicated in the signaling pathways of RELN. The cytogenetic location of this gene is 7q22. With this background, we aimed to observe the mRNA expression levels of RELN gene in patients with schizophrenia [8].

## Materials and methods

This study was performed at the Department of Biochemistry in collaboration with the Department of Psychiatry and Genetics Laboratory at BLDE (Deemed to be University), Shri B. M. Patil Medical College, Hospital and Research Centre, Vijayapura. The biochemical and genetic association analysis of RELN gene markers with neurodevelopmental disorders such as schizophrenia, major depressive disorder, and bipolar disorder is a part of our current work. In this analytical, cross-sectional study, 108 samples with 54 cases (study group) and 54 controls (control group) were analyzed over six months (samples were collected from September 2022 to August 2023). All the subjects were in the age range of 20-65 years. After explaining the study to the patients, their voluntary consent was taken in writing. The research data and the privacy of the participants were protected according to the institutional ethical committee guidelines. The study was approved by the BLDE (Deemed to be University) Institutional Ethical Committee [Approval number: BLDE (DU)/IEC/718/2022-23].

Inclusion criteria

Patients diagnosed with schizophrenia with standard criteria based on DSM V and International Classification of Disease 10 (ICD-10) were included in this study.

Exclusion criteria

We excluded cases of depressive disorder, schizoaffective disorder, and bipolar disorder. We also excluded schizophrenic symptoms secondary to alcohol, cannabis, or other drug-induced disorders.

Sample collection

Blood samples were collected from all subjects in different tubes: 2 ml in a fluoride vacutainer (Becton, Dickinson and Company, New Jersey, USA), 2 ml in a plain vacutainer (Becton, Dickinson and Company, New Jersey, USA), and 1 ml in an ethylenediaminetetraacetic acid (EDTA) vacutainer (Becton, Dickinson and Company, New Jersey, USA) as per the parameter to be assayed. The serum was separated within one hour by centrifugation of the blood sample at 3000 rpm for 15 minutes and then the supernatant (serum) was used for the estimation of various parameters and stored in the deep freezer at -800°C.

Anthropometrical data

Anthropometrical data such as height (feet), weight (kg), body mass index (BMI; kg/m^2^), waist circumference (WC; inches), and physiological data such as systolic blood pressure systolic (SBP; mmHg) and diastolic blood pressure (DBP; mmHg) were recorded.

BMI

Weight was recorded to the nearest kilogram (kg) with the subject standing on the weighing machine without shoes and normal clothing. Height was measured with the subject standing upright, barefooted, feet together, back and heels against the upright bar of the height scale, with the head upright in a horizontal plane in the "look straight ahead" position.

Blood pressure (BP)

The blood pressure was measured by a mercurial sphygmomanometer (mmHg).

Biochemical analysis

For the estimation of blood glucose, random samples of blood were collected in a sodium fluoride vacutainer tube. For the estimation of the lipid profile-related parameters, random blood samples were collected in a plain vacutainer tube without anticoagulant. For the molecular analysis, the samples were collected in the EDTA vacutainer. All the specimens were immediately subjected to assays for blood glucose and lipid profile analysis. The tests were carried out on Mispa Viva Semi Automated Clinical Chemistry Analyzer (Agappe Diagnostics Ltd., Kerala, India).

Blood Glucose

The test was programmed and carried out in the semi auto analyser using standard kits (Agappe Diagnostics Ltd., Kerala, India). 

Lipid Profile

Lipid profile was estimated to know the amount of total cholesterol (TC) in the blood and also measured the level of high-density lipoprotein-cholesterol (HDL-C), serum triglycerides (TGs), and serum low-density lipoprotein-cholesterol (LDL-C).

RNA isolation and synthesis of complementary DNA (cDNA)

A blood sample of 300 µl was used to extract RNA by using nucleospin RNA extraction kit (MACHEREY-NAGEL GmbH & Co. KG, Dueren, Germany). RNA was measured in 260/280 nm and pure RNA was measured in ng/ml. All the samples were of 0.5 to 5mg concentration. cDNA synthesis performed by using High-Capacity cDNA Reverse Transcription Kit (Applied Biosystems^TM^, California, USA). A total of 1 µg of RNA was reverse transcribed.

Quantitative real-time PCR

Real-time reverse transcription-polymerase chain reaction (RT-PCR) was performed on a fluorescence thermal cycler (Quant Studio™ 5 Real-Time PCR System, Applied Biosystems^TM^, California, USA). A standard two-step procedure was applied. RNAs were reverse transcribed into single strand cDNAs using oligo(dT) primers and Moloney-Murine Leukemia Virus (M-MLV) reverse transcriptase (Qiagen, Maryland, USA). Real-time RT-PCR was performed in 25 μL reaction mixtures consisting of cDNA, 0.5 μM specific primer sets for each target gene, and SYBR Green PCR Master Mix (PowerUp™ SYBR™ Green Master Mix, Applied Biosystems^TM^, California, USA). The conditions were 50O°C for two minutes, 95O°C for 10 minutes, followed by 40 repetitive cycles of 95O°C for 15 seconds, and 60O°C for one minute followed by five minutes elongation at 72O°C. β-actin was used as an internal control to normalize for initial RNA input and this gene was found to display remarkably stable expression levels across experimental treatments. Primers of RELN gene for RT-PCR were 5’-CATGGTTGCAAGTGTGACCC-3’ and 5’-AAACCAGGGCCTTACCACTG-3’ and the primers of β-actin were F: 5-AAGATCATTGCTCCTCCTGAGC-3 and R: 5-TCCTGCTTGCTGATCCACATC-3.

Statistical analysis

Statistical analysis was carried out by using IBM SPSS Statistics for Windows, Version 20 (Released 2011; IBM Corp., Armonk, New York, United States) and all the results are mentioned as mean ± standard deviation. An independent sample-t test was performed to compare the mRNA expression levels and p-value <0.05 was considered as statistically significant. Mann-Whitney U and Spearman's rho correlation coefficient tests were performed to compare other parameters between the study and the control groups.

## Results

The study involved 108 subjects, out of which 54 were patients with schizophrenia while 54 were healthy controls. In the study group, 29 were female (53.7%) and 25 were male (46.3%) patients whereas in control group, 27 were female (50%) and 27 were male (50%) subjects (Table 1).

**Table 1:** Number of subjects in the study and control groups

Gender	Cases		Control	
	No. of patients	Percentage	No. of patients	Percentage
Female	29	53.7	27	50.0
Male	25	46.3	27	50.0
Total	54	100.0	54	100.0

The mean ± standard deviation and the significance level of each parameter were analyzed by the Mann-Whitney U test in both groups. There was no difference in both groups in terms of the age group of the subjects, and the systolic and diastolic blood pressures. BMI and WC were significantly greater in the study group than in the control group (Table 2).

**Table 2:** Clinical and demographic profile of the subjects in both the study and control groups ***p<0.001 very highly significant; BMI, Body mass index; WC, Waist circumference; BP, Blood pressure.

Parameters	Study group (n= 54)	Control group (n=54)	Mann-Whitney U test value	p-value
Age (years)	40.83±9.931	44.46±10.914	1265	0.071
Weight (kg)	59.65±17.13	58.03±10.41	1163	0.428
Height (cm)	148.81±6.83	155.61±8.85	496	<0.001***
BMI (kg/m^2^)	27.45±5.60	24.03±3.60	428	<0.001***
WC (cm)	81.93±13.10	60.92±9.34	341.5	<0.001***
Systolic BP (mmHg)	123.02±16.22	124.29±9.88	1422	0.578
Diastolic BP (mmHg)	83.61±9.394	81.79±9.167	1374.5	0.387

Table 3 reflects the significant increase in RBS in the study group compared to the control group (p<0.001). Lipid indices like serum TC and LDL-C were significantly greater in the study group vs. the control group (p<0.001), whereas the HDL-C levels were significantly lower in the study group compared to the control. The study group also exhibited increased TG levels but they were not statistically significant. The significantly higher lipid ratios in the study group indicated that the patients suffering from psychotic disorders are more prone to developing metabolic syndrome as well as cardiovascular disease in the future.

**Table 3:** Biochemical parameters in the study and control groups ***p<0.001 highly significant; RBS, Random blood sugar; TC, Total cholesterol; TG, Triglycerides; HDL-C, High-density lipoprotein–cholesterol; LDL-C, Low-density lipoprotein-cholesterol; VLDL-C, Very low-density lipoprotein-cholesterol.

Parameters	Study group (n= 54)	Control group (n=54)	Mann-Whitney U test value	p-value
RBS (mg/dL)	147.02±37.368	123.59±18.278	886	0.001***
TC (mg/dL)	223.11±46.683	167.23±16.739	312.5	0.001***
Serum TG (mg/dL)	155.94±66.873	131.54±20.732	1343	0.312
Serum direct HDL-C (mg/dL)	25.85±7.181	45.88±6.284	76.5	0.001***
Serum LDL-C (mg/dL)	167.41±48.919	95.79±13.309	232	0.001***
Serum VLDL-C (mg/dL)	31.81±13.461	26.77±5.281	1315	0.239
TG/HDL-C ratio (mg/dL)	4.9475±2.46939	2.9046±0.53115	301	0.001***
LDL/HDL-C ratio (mg/dL)	4.4102±2.24474	2.1284±0.40831	402.5	0.001***
TC/HDL-C ratio (mg/dL)	6.2504±2.79128	3.6772±0.44596	395.5	0.001***

Table 4 shows a significant positive correlation between BMI and biochemical parameters such as RBS and lipid profile levels, and a negative correlation between lipid ratios and BMI (BMI vs. TG/HDL-C r : -0.090, p : 0.515; BMI vs. LDL-C/HDL-C r : -0.092, p : 0.507; BMI vs. CHOL/HDL-C r : -0.054, p : 0.701).

**Table 4:** Bivariate correlation between BMI and biochemical parameters in the study group ***p<0.001, very highly significant; RBS, Random blood sugar; TC, Total cholesterol; TG, Triglycerides; HDL-C, High-density lipoprotein–cholesterol; LDL-C; Low-density lipoprotein-cholesterol; VLDL-C, Very low-density lipoprotein-cholesterol; BMI, Body mass index.

Biochemical parameters	BMI	
	Spearman's rho correlation coefficient (r) value	p-value
RBS (mg/dL)	-0.011	0.957
Serum TC (mg/dL)	0.112	0.587
Serum TG (mg/dL)	0.010	0.960
Direct HDL-C (mg/dL)	0.096	0.642
Serum LDL-C (mg/dL)	0.128	0.534
Serum VLDL-C (mg/dL)	0.003	0.990
BMI vs. TG/HDL-C	-0.090	0.515
BMI vs. LDL-C/HDL-C	-0.092	0.507

**Figure 1:**
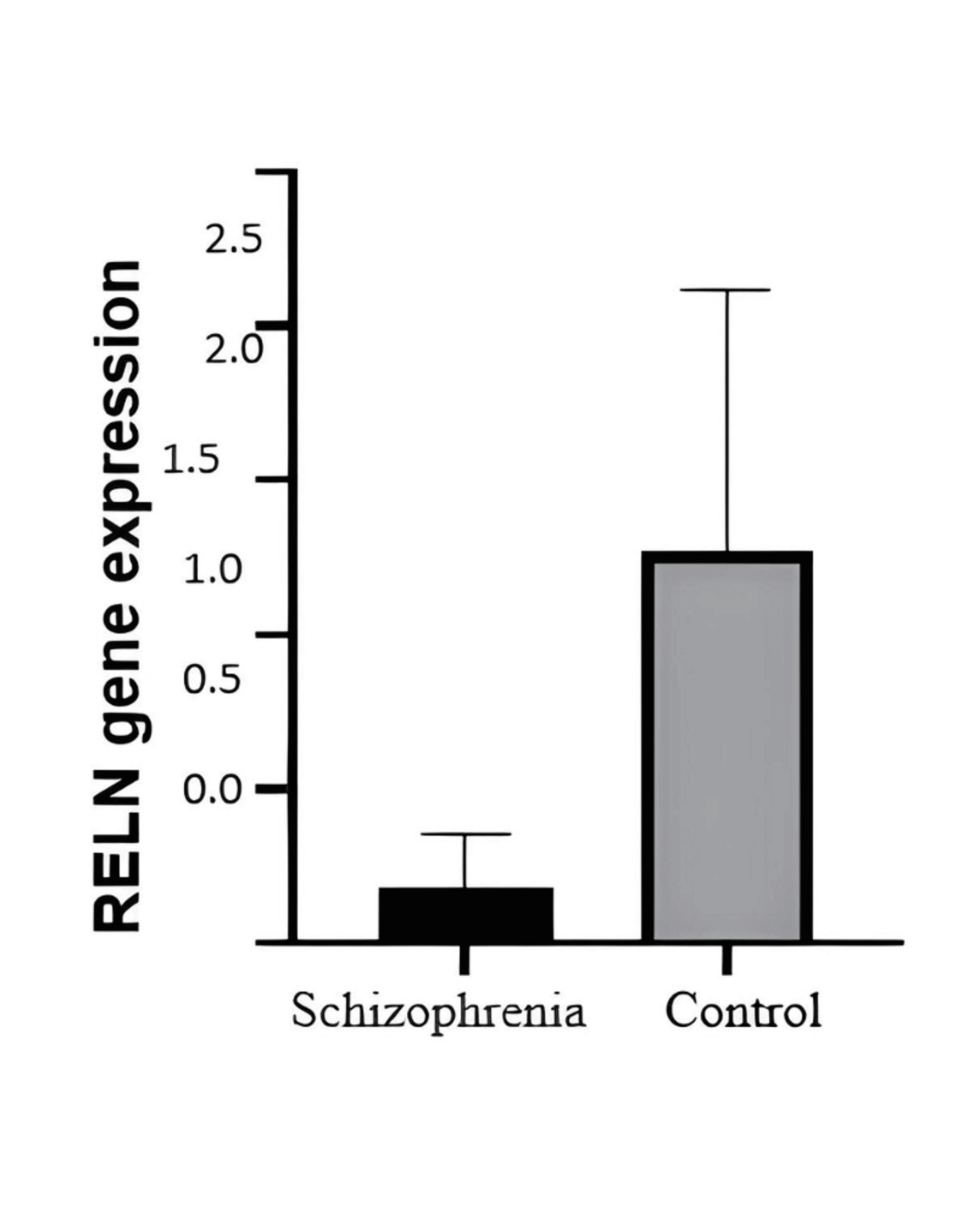
Bar graph showing mRNA expression of the down regulated RELN gene by quantitative real-time PCR in response to schizophrenia against the sample from the control group PCR, Polymerase chain reaction

RT-PCR results revealed a decrease in RELN gene expression in the study group with schizophrenia patients vs. the control group (Figure 1). The age group of the participants in the expression study was from 20 to 65 years. 

The delta-delta Ct expression level was significantly positively correlated with BMI, while WC, blood glucose, lipid profile (except for HDL-C level), and lipid ratios were significantly negatively correlated with it (Table 5).

**Table 5:** Bivariate correlation between RELN gene expression level with BMI, WC and other biochemical parameters in the study group (patients with schizophrenia) ***p<0.001 very highly significant; BMI, Body mass index; WC, Waist circumference; RBS, Random blood sugar; TC, Total cholesterol; TG, Triglycerides; HDL-C, High-density lipoprotein-cholesterol; LDL-C; Low-density lipoprotein-cholesterol; VLDL-C, Very low-density lipoprotein-cholesterol; DDCT, Delta delta Ct.

Parameters	RELNgeneExpression	
	r-Value	p-value
BMI (kg/m^2^)	0.345	0.085
WC (cm)	-0.141	0.491
RBS (mg/dl)	-0.084	0.676
Serum TC (mg/dl)	-0.319	0.128
Serum TG (mg/dl)	-0.373	0.073
Direct HDL-C (mg/dl)	0.377	0.070
Serum LDL-C (mg/dl)	-0.717	0.000
Serum VLDL-C (mg/dl)	-0.364	0.067
DDCT vs. TG/HDL-C	-0.690	0.000
DDCT vs. LDL-C/HDL-C	-0.917	0.000
DDCT vs. TC/HDL-C	-1.000^***^	0.000

## Discussion

Schizophrenia is a chronic mental disorder characterized by a decline in the patient’s ability to think, feel, and behave. It is a complex condition that can cause various symptoms, including delusions, disorganized speech, hallucinations, strange behavior, and negative symptoms such as social withdrawal and lack of motivation. The global prevalence rate of schizophrenia is estimated to be approximately 1%. However, it varies widely across different regions and countries, with some studies reporting rates as high as 3% in certain populations [9]. The incidence of schizophrenia is estimated to be around 0.4 to 0.6 per 1000 people per year [10].

In this study, 50% of the participants in the age range of 20 to 65 years suffered from schizophrenia. Based on the DSM-V and ICD-10 criteria, they mainly suffered from positive and negative symptoms and showed moderate severity on the BPRS Scale. The anthropometric parameters analyzed in these participants showed highly significant differences compared to the control group. Moreover, the RELN gene expression analysis in the study group showed a decrease in expression compared to the controls.

The protein produced by the RELN gene is essential for the formation of neurons and their migration during embryonic development. It exerts several important functions in the brain including the regulation of neuronal migration, dendritic growth and branching, dendritic spine formation, synaptogenesis, and synaptic plasticity. Previous studies have suggested that there may be a link between the RELN gene and schizophrenia. Specifically, it has been found that individuals with schizophrenia may have lower levels of RELN in some areas of the brain, such as the prefrontal cortex and hippocampus. Additionally, genetic variations impacting the RELN gene expression are associated with an increased risk of schizophrenia development [11]. According to other research, individuals with schizophrenia may have changes in their lipid profile, including lower levels of HDL-C and changes in the concentrations of TGs, LDL-C, and TC [12]. Another study observed that 40% percent of the studied patient population, without a previous history of diabetes, had newly diagnosed disturbances of glucose metabolism [impaired glucose tolerance (IGT) or type 2 diabetes]. The prevalence of diabetes in patients with schizophrenia was much higher than the prevalence of diabetes in the general population [13]. In our study, RBS varied between the two groups with an increased concentration in the patients with schizophrenia (p<0.001).

A group of researchers from Nanjing Medical University, Wuxi, China performed association studies in the Chinese population to investigate the genetic correlation of RELN with schizophrenia. They showed that RELN expression was decreased in the blood of untreated patients with schizophrenia [14]. These findings are similar to other studies showing that RELN gene expression is not observed in the brain and blood of patients with schizophrenia. Another study also showed that 12 weeks of antipsychotic therapy dramatically increased the expression level of RELN mRNA in these patients. There are no association studies related to RELN in Indian patients with schizophrenia. Therefore, our primary objective was to evaluate this association in an Indian population. In another study, patients with schizophrenia showed greater peripheral blood methylation levels in the RELN gene promoter than healthy controls. It was demonstrated that RELN methylation levels were linked to worse cognitive performance, specifically in working memory and attention [15].

Similarly, we found that RELN gene mRNA expression was downregulated in the study group compared to control group because of antipsychotic medications. Our results showed that RELN mRNA expression levels were not statistically significant in patients with schizophrenia after antipsychotic treatment.

Brietzke et al. (2018) observed that BMI may be linked to RELN pathway dysregulation. Our study showed a correlation between BMI and RELN gene expression in the study group vs. the control group [16]. According to Hable et al. (2009), RELN expression is decreased in the left prefrontal cortex in patients with chronic schizophrenia. This finding suggests that the dysregulation of RELN expression may contribute to the pathophysiology of schizophrenia. Its reduced expression in the prefrontal cortex of chronic schizophrenia patients may contribute to this disorder’s cognitive and behavioral deficits [17]. In addition, other studies have demonstrated that RELN gene plays a key role in regulating dopamine signaling. Our study also showed that dysregulation of RELN expression may contribute to the pathophysiology of schizophrenia [18]. Moreover, Gupta et al. (2013) estimated lipid and glucose parameters in patients with schizophrenia and showed significant changes in LDL-C, glucose, TGs, and HDL-C in those on quetiapine, risperidone, and olanzapine [19].

Apart from gene expression, other factors of cardiovascular disease such as dyslipidemia, obesity, metabolic syndrome, diabetes, and physical inactivity significantly increase with the antipsychotic treatment in patients with schizophrenia and are strongly associated with the condition. Overall, the expression profile of the RELN gene in schizophrenia has shed light on the gene function in its pathophysiology. However, more research should be carried out to understand the complex mechanisms underlying the relationship between RELN and schizophrenia.

Study limitations

The sample size used for this study was small. The RELN gene analysis should have been done at the onset of schizophrenia and after treatment. The effect of the dose and duration of the antipsychotic drug treatment must also be considered to establish a possible link between the drugs used and metabolic syndrome.

## Conclusions

Schizophrenia is a devastating neuropsychiatric condition of uncertain etiology with significant adverse effects on affected people, their families, and society. A heterogeneous population is seen in India with a high degree of inbreeding. This makes it necessary to screen many patients, perhaps within each age group, in order to get a true picture of the contribution of RELN mRNA expression to schizophrenia. To know the association of mRNA expression and mutations in genes related to schizophrenia, research should be carried out in larger populations for a future perspective. Studies have reported that downregulation of the RELN gene expression makes a patient prone to psychotic disorders, including schizophrenia and antipsychotic drugs upregulate the gene. However, we found a decreased expression of the RELN gene in patients who were undergoing treatment with antipsychotic drugs. This may be because of the limited sample size of this study. Hence, the exact role of the RELN gene in the pathophysiology of schizophrenia cannot be derived. Further studies are necessary to evaluate the role of other genes such as DISC1 (DISC1 scaffold protein), AGO2 (argonaute RISC catalytic component 2), RUNX3 (RUNX family transcription factor 3), SIGIRR (single Ig and TIR domain containing), SLC18A1 (solute carrier family 18 member A1), NRG1 (neuregulin 1), CHRNB2 (cholinergic receptor nicotinic beta 2 subunit), PRKAB2 (protein kinase AMP-activated non-catalytic subunit beta 2), LDB1 (LIM domain binding 1), and ZNF74 (zinc finger protein 74) that could contribute to the development of neurological disorders in Indian families with susceptibility to schizophrenia. Lifestyle changes and regular monitoring of patients on antipsychotic drug treatment are necessary to improve their quality of life. Additionally, therapeutic interventions that decrease lipid abnormalities will also help in improving the life expectancy of patients with schizophrenia.
